# Allied health – 3007. The biomarker of sublingual immune therapy

**DOI:** 10.1186/1939-4551-6-S1-P183

**Published:** 2013-04-23

**Authors:** Masafumi Sakashita, Yoshimasa Imoto, Yoko Osawa, Noboru Takahashi, Seita Kubo, Hideyuki Yamamoto, Takechiyo Yamada, Shigeharu Fujieda

**Affiliations:** 1 The Departments of Otorhinolaryngology Head & Neck Surgery, University of Fukui, Fukui, Japan

## Background

Allergic rhinitis (AR) is recognized as a major health problem worldwide. Allergen-specific immunotherapy (SIT) is the only available treatment that can alter the natural course of allergic disease. Recent findings in experimental models of allergic rhinitis suggest that complement 3a and 5a regulate the development of maladaptive Th2 and Th17 immunity. We investigated the changes of C3a, C5a, IL-17a in the serum of patients treated by Sublingual immune therapy (SLIT).

## Methods

Symptoms were recorded in the allergy diary. The total symptom medication scores were calculated based on each symptoms and medication. We measured the C3a, C5a, IL-17a levels in serum of the 20 identical subjects by ELISA during 5 years.

## Results

C3a and IL-17a showed significant decrease year by year during 5 years (p < 0.01).

## Conclusions

C3a and IL-17a can be objective biomarker for following up the patients of SLIT.

